# Supplementary material for: Integrating complementary datasets for constructing microbiome–metabolome–disease pathways
Source: Bioinformatics. 2026 Oct 20;42(11):btag787. doi: 10.1093/bioinformatics/btag787 (PMC13725751; doi:10.1093/bioinformatics/btag787)
Supplement: Supplementary file 1 [file btag787_supplementary_data.pdf]

Supplement for “Integrating complementary datasets for constructing microbiome-metabolome-disease pathways"

October 16, 2026

1 Additional Simulation Results

1.1 Sensitivity analysis of different signal strengths

Here, we added additional simulation results by changing the signal strength through varying the σ_δ in model (1) and σ_ε in model (2). Specifically, we set $\sigma_\delta = \sigma_\varepsilon = 3$ to obtain a R^2 about 34% in model (1) and 15% in model (2) when $\theta^* = 0$. We also set $\sigma_\delta = \sigma_\varepsilon = 4$ to obtain a R^2 about 16% in model (1) and 6% in model (2) when $\theta^* = 0$. The rest parameters are the same as the simulation setting in the main text (page 6). We show their results in Figure S1 and Figure S2, respectively. We also present a summary of Type I error, power when $\theta^* = 0.6$, and AUC of selection of $\boldsymbol{\gamma}^0$ and $\boldsymbol{\beta}^0$ under different σ_δ and σ_ε in Table S1, Table S2 and Table S3, respectively. As expected, increasing the R^2 yields higher power for testing θ^* while maintaining proper Type I error control across all settings. It also improves selection performance, as reflected by higher AUC values of selection for $\boldsymbol{\gamma}^0$ and $\boldsymbol{\beta}^0$.

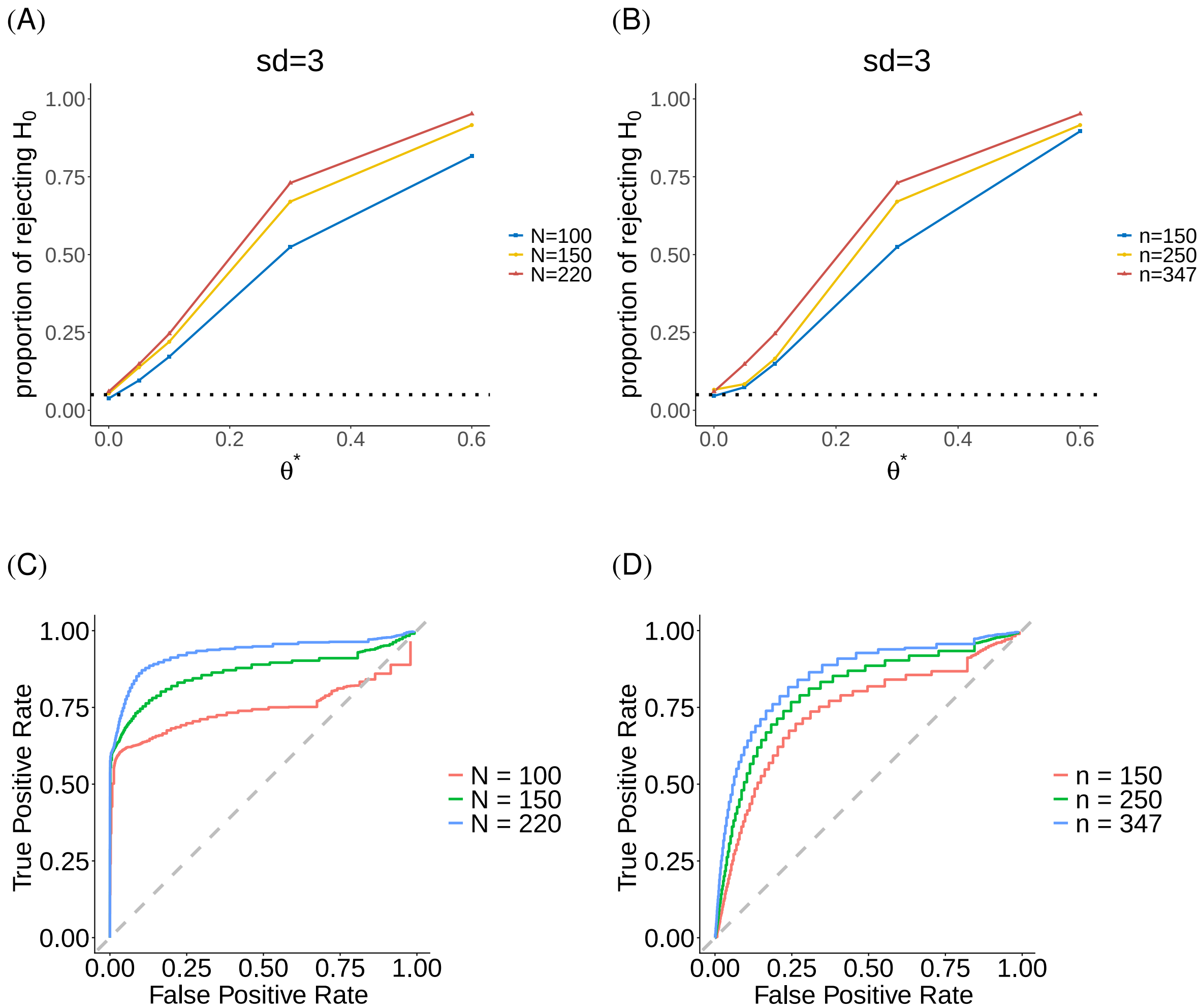

Figure S1: The performance of proposed method when $\sigma_\delta = \sigma_\varepsilon = 3$. Type-I error and power for testing $H_0 : \theta^* = 0$ under varying sample sizes. (A) External sample size varies; (B) Target sample size varies. The horizontal dotted line indicates the nominal level of 0.05. (C) ROC curves for γ^0 under different external sample sizes, with AUCs of 0.732, 0.862, and 0.921 for $N = 100$, 150, and 220, respectively. (D) ROC curves for $\boldsymbol{\beta}^0$ under different target sample sizes, with AUCs of 0.736, 0.799, and 0.843 for $n = 150$, 250, and 347, respectively.

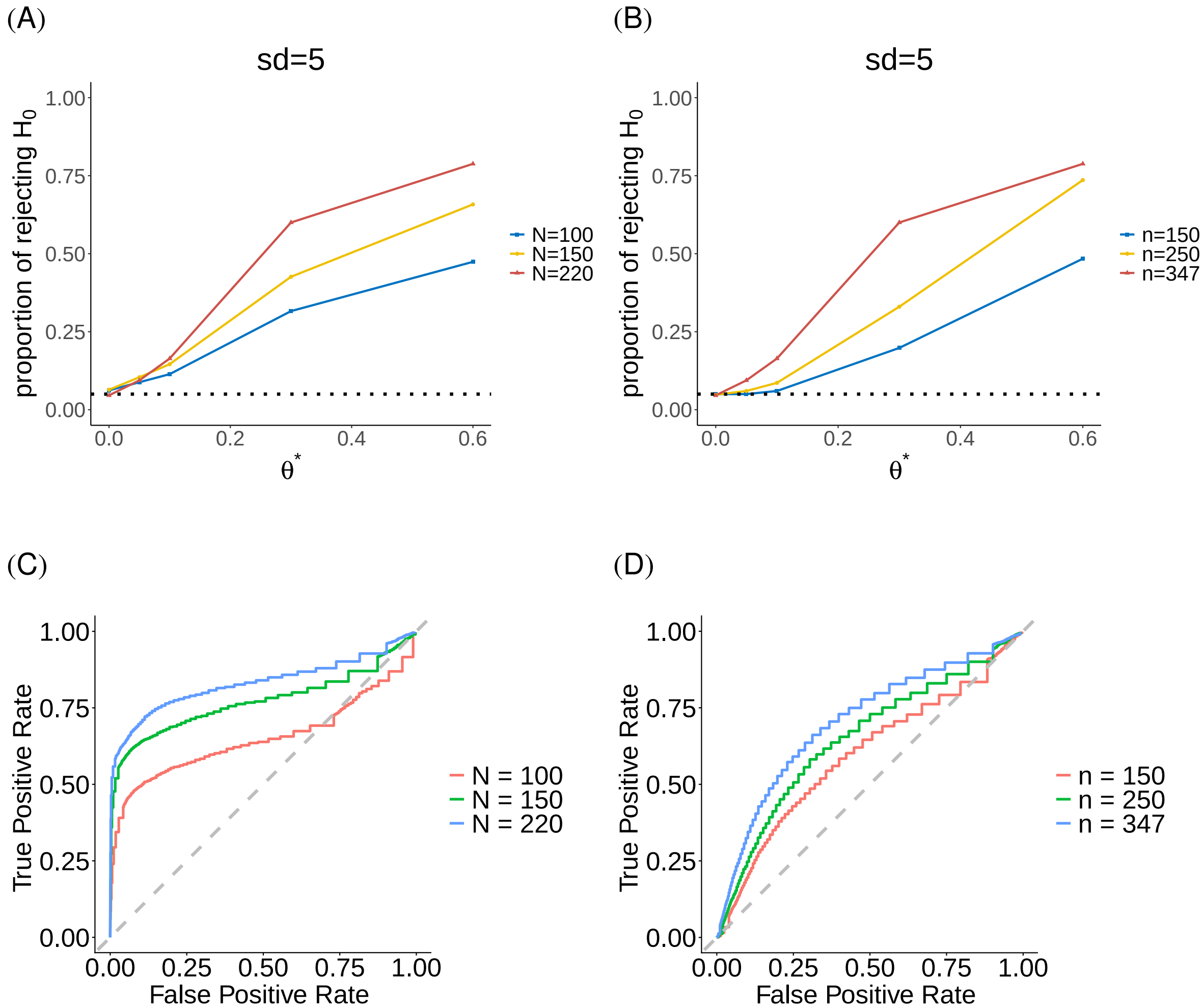

Figure S2: The performance of proposed method when $\sigma_\delta = \sigma_\varepsilon = 5$. Type-I error and power for testing $H_0 : \theta^* = 0$ under varying sample sizes. (A) External sample size varies; (B) Target sample size varies. The horizontal dotted line indicates the nominal level of 0.05. (C) ROC curves for γ^0 under different external sample sizes, with AUCs of 0.647, 0.776, and 0.831 for $N = 100$, 150, and 220, respectively. (D) ROC curves for β^0 under different target sample sizes, with AUCs of 0.603, 0.654, and 0.708 for $n = 150$, 250, and 347, respectively.

sample size setting	Type I error		
	$\sigma_\delta = \sigma_\varepsilon = 3$	$\sigma_\delta = \sigma_\varepsilon = 4$	$\sigma_\delta = \sigma_\varepsilon = 5$
external sample size ($n = 347$ fixed)			
$N = 100$	0.038	0.058	0.062
$N = 150$	0.054	0.049	0.064
$N = 220$	0.060	0.048	0.046
target sample size ($N = 220$ fixed)			
$n = 150$	0.046	0.044	0.050
$n = 250$	0.066	0.060	0.048

Table S1: The Type I error of testing $H_0 : \theta^* = 0$ under different sample size, σ_δ, and σ_ε.

sample size setting	Power($\theta^* = 0.6$)		
	$\sigma_\delta = \sigma_\varepsilon = 3$	$\sigma_\delta = \sigma_\varepsilon = 4$	$\sigma_\delta = \sigma_\varepsilon = 5$
external sample size ($n = 347$ fixed)			
$N = 100$	0.816	0.602	0.474
$N = 150$	0.916	0.718	0.658
$N = 220$	0.952	0.886	0.788
target sample size ($N = 220$ fixed)			
$n = 150$	0.896	0.712	0.484
$n = 250$	0.948	0.872	0.736

Table S2: The power of testing $H_0 : \theta^* = 0$ under different sample size, σ_δ, and σ_ε.

sample size setting	AUC		
	$\sigma_\delta = \sigma_\varepsilon = 3$	$\sigma_\delta = \sigma_\varepsilon = 4$	$\sigma_\delta = \sigma_\varepsilon = 5$
stage 1 selection of $\boldsymbol{\gamma}^0$ ($n = 347$ fixed)			
$N = 100$	0.732	0.687	0.647
$N = 150$	0.862	0.815	0.776
$N = 220$	0.921	0.870	0.831
stage 2 selection $\boldsymbol{\beta}^0$ ($N = 220$ fixed)			
$n = 150$	0.736	0.656	0.603
$n = 250$	0.799	0.718	0.654
$n = 347$	0.843	0.771	0.708

Table S3: The AUC of selection for $\boldsymbol{\gamma}^0$ and $\boldsymbol{\beta}^0$ under different sample sizes, σ_δ, and σ_ε.

1.2 Sensitivity analysis of threshold in stability selection

Here, we examine the impact of choice of threshold in stability selection by using additional threshold of 0.6, 0.7, and 0.9 across different sample size settings when $\sigma_\delta = \sigma_\varepsilon = 4$, the rest settings are the same as in the main text (page 6). We plot the ROC curve in the Figure S3 below. From the ROC curve and the AUCs from each sample size setting, we conclude that the results seems very robust to the choice of threshold.

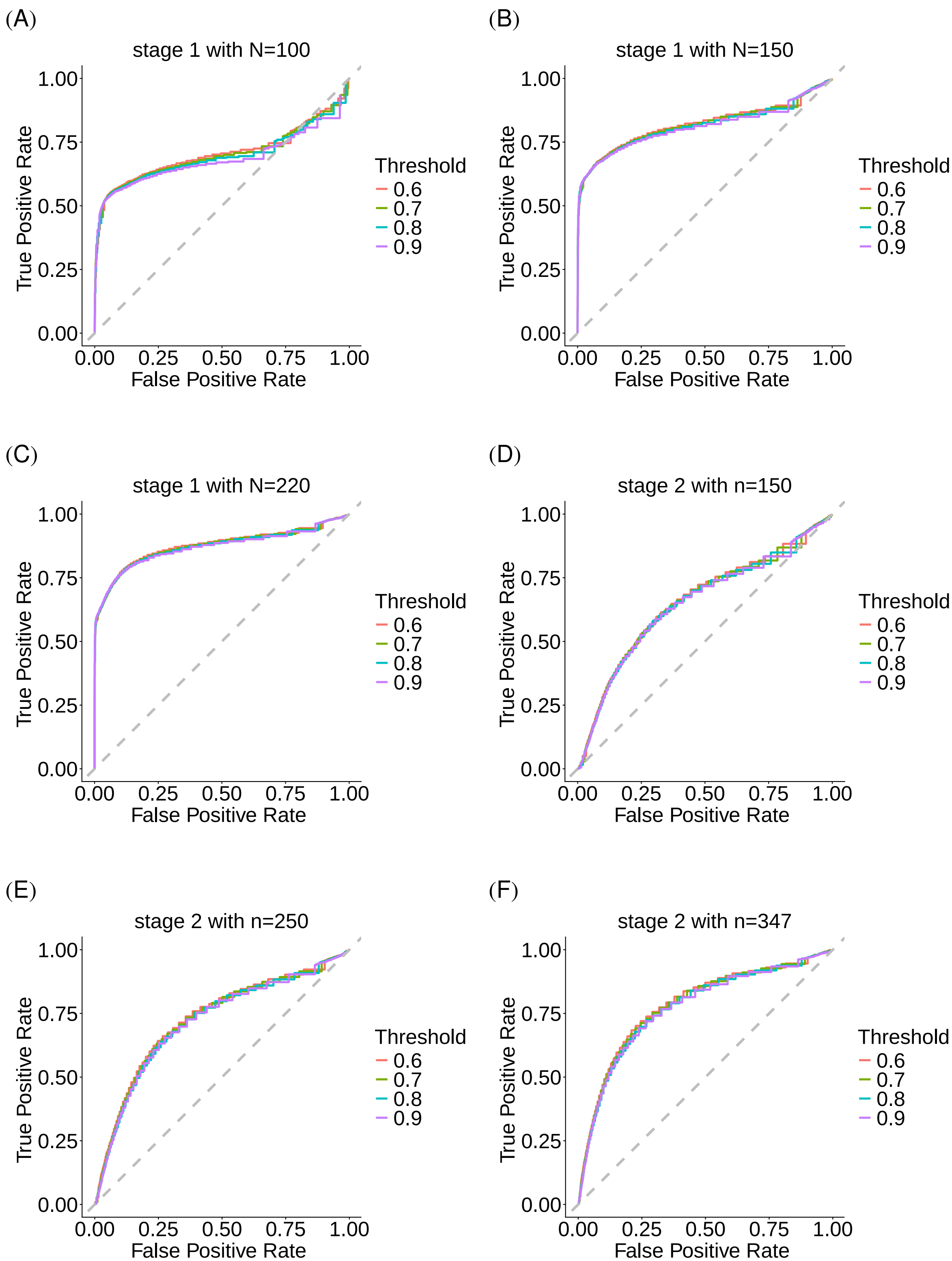

Figure S3: (A) ROC curves for γ^0 with external sample sizes of 100, under different thresholds of 0.6, 0.7, 0.8 and 0.9, and their AUCs of 0.708, 0.700, 0.687 and 0.657, respectively. (B) ROC curves for γ^0 with external sample sizes of 150, under different thresholds of 0.6, 0.7, 0.8 and 0.9, and their AUCs of 0.825, 0.821, 0.815 and 0.806, respectively. (C) ROC curves for β^0 with target sample sizes of 220, under different thresholds of 0.6, 0.7, 0.8 and 0.9, and their AUCs of 0.877, 0.873, 0.870 and 0.865, respectively. (D) ROC curves for β^0 with target sample sizes of 150, under different thresholds of 0.6, 0.7, 0.8 and 0.9, and their AUCs of 0.666, 0.661, 0.656 and 0.649, respectively. (E) ROC curves for β^0 with target sample sizes of 250, under different thresholds of 0.6, 0.7, 0.8 and 0.9, and their AUCs of 0.733, 0.727, 0.718 and 0.712, respectively. (F) ROC curves for β^0 with target sample sizes of 347, under different thresholds of 0.6, 0.7, 0.8 and 0.9, and their AUCs of 0.786, 0.780, 0.771 and 0.766, respectively.

1.3 Inflated Type I error when no penalty on θ

Here, we clarify our choice of penalizing both $\boldsymbol{\beta}$ and θ instead of penalizing $\boldsymbol{\beta}$ only from both methodological and numerical perspectives. We first considered the choice of penalizing $\boldsymbol{\beta}$ only in the second-stage model. Specifically, the estimator is obtained by minimizing the following objective function:

$$(\widehat{\boldsymbol{\beta}}_{\text{single}}, \widehat{\theta}_{\text{single}}) = \underset{\boldsymbol{\beta},\theta}{\operatorname{argmin}} \left\{ \frac{1}{2n} \|\mathbf{y} - \mathbf{X}\widehat{\boldsymbol{\gamma}}\theta - \mathbf{X}\boldsymbol{\beta}\|_2^2 + \lambda \|\boldsymbol{\beta}\|_1 \right\}, \tag{1}$$

where $\mathbf{y} = (y_1, \ldots, y_n)^\intercal$, $\mathbf{X} = (\mathbf{x}_1, \ldots, \mathbf{x}_n)^\intercal$, and λ is the tuning parameter.

From (1), the KKT condition for θ is given by

$$(\mathbf{X}\widehat{\boldsymbol{\gamma}})^\intercal \mathbf{r} = 0, \tag{2}$$

where $\mathbf{r} = \mathbf{y} - \mathbf{X}\widehat{\boldsymbol{\gamma}}\hat{\theta}_{\text{single}} - \mathbf{X}\widehat{\boldsymbol{\beta}}_{\text{single}}$. The KKT condition for $\boldsymbol{\beta}$ yields

$$\mathbf{X}^\intercal \mathbf{r} = n\lambda \mathbf{s}, \tag{3}$$

where $\mathbf{s} = (s_1, \ldots, s_p)^\intercal$ and

$$s_j = \begin{cases} \text{sign}(\hat{\beta}_{\text{single},j}), & \hat{\beta}_{\text{single},j} \neq 0 \\ \in [-1, 1], & \hat{\beta}_{\text{single},j} = 0 \end{cases}.$$

Combining (2) and (3) yields

$$\widehat{\boldsymbol{\gamma}}^\intercal \mathbf{s} = 0, \tag{4}$$

where $\mathbf{s}$ denotes the subgradient associated with the ℓ_1 penalty on $\boldsymbol{\beta}$.

Equation (4) shows that the support and signs of $\widehat{\boldsymbol{\beta}}$ are subject to an additional orthogonality constraint determined by $\widehat{\boldsymbol{\gamma}}$. Since $\widehat{\boldsymbol{\gamma}}$ is also sparse, this condition restricts the feasible sign patterns of $\widehat{\boldsymbol{\beta}}$ beyond those imposed by the ℓ_1 penalty alone. In particular, when a true signal $\beta_j \neq 0$ corresponds to a variable that lies in the support of $\widehat{\boldsymbol{\gamma}}$, the orthogonality constraint may prevent β_j from being selected, even if it is associated with the outcome. Consequently, penalizing only $\boldsymbol{\beta}$ can introduce additional false negatives in variable selection.

In contrast, since we penalize both $\boldsymbol{\beta}$ and θ as follows:

$$(\widehat{\boldsymbol{\beta}}, \hat{\theta}) = \underset{\boldsymbol{\beta},\theta}{\operatorname{argmin}} \left\{ \frac{1}{2n} \|\mathbf{y} - \mathbf{X}\hat{\boldsymbol{\gamma}}\theta - \mathbf{X}\boldsymbol{\beta}\|_2^2 + \lambda_\beta (\|\boldsymbol{\beta}\|_1 + |\theta|) \right\}, \tag{5}$$

the KKT condition for θ is

$$(\mathbf{X}\hat{\boldsymbol{\gamma}})^\intercal \mathbf{z} = n\lambda_\beta s_\theta, \tag{6}$$

where $\mathbf{z} = \mathbf{y} - \mathbf{X}\widehat{\boldsymbol{\gamma}}\hat{\theta} - \mathbf{X}\widehat{\boldsymbol{\beta}}$ and

$$s_\theta = \begin{cases} \text{sign}(\hat{\theta}), & \hat{\theta} \neq 0 \\ \in [-1, 1], & \hat{\theta} = 0 \end{cases}.$$

The KKT condition for $\boldsymbol{\beta}$ yields that

$$\mathbf{X}^\intercal \mathbf{z} = n\lambda_\beta \mathbf{t}, \tag{7}$$

where $\mathbf{t} = (t_1, \ldots, t_p)^\intercal$, $t_j = \begin{cases} \text{sign}(\hat{\beta}_j), & \hat{\beta}_j \neq 0 \\ \in [-1, 1], & \hat{\beta}_j = 0 \end{cases}$. Combining (6) and (7), we have

$$\widehat{\boldsymbol{\gamma}}^\intercal \mathbf{t} = s_\theta. \tag{8}$$

Compared with (4), the KKT condition in (8) is less restrictive. When only $\boldsymbol{\beta}$ is penalized, the subgradient associated with $\boldsymbol{\beta}$ must satisfy the exact orthogonality condition $\widehat{\boldsymbol{\gamma}}^\intercal \mathbf{s} = 0$. In contrast, when both $\boldsymbol{\beta}$ and θ are penalized, the additional subgradient s_θ allows the inner product $\widehat{\boldsymbol{\gamma}}^\intercal \mathbf{t}$ to deviate from zero. Consequently, the feasible set of subgradients for $\boldsymbol{\beta}$ is enlarged, and the sign pattern of the selected coefficients is no longer required to satisfy a strict orthogonality constraint with $\widehat{\boldsymbol{\gamma}}$. This additional flexibility reduces the chance that true signals in $\boldsymbol{\beta}$, particularly those overlapping with or highly correlated with $\mathbf{X}\widehat{\boldsymbol{\gamma}}$, are excluded solely because of the KKT constraint. The discussion above focuses on the effect of penalizing θ on the selection of $\boldsymbol{\beta}$. This distinction also carries over to the inference of θ^*. In our framework, the selected $\widehat{\boldsymbol{\beta}}$ is a key input to the subsequent permutation-based inference procedure. Therefore, any difference in the selection of $\boldsymbol{\beta}$ may affect the construction of the test statistic and, consequently, the inference for θ^*. To illustrate the impact on inference, we conducted an additional simulation study comparing the proposed approach with the alternative that penalizes only $\boldsymbol{\beta}$. The simulation settings were identical to those in the main text, with $\sigma_\delta = \sigma_\varepsilon = 4$. Figure S4 shows that, when θ is not penalized, the empirical Type I error is substantially inflated across all sample size settings, ranging from approximately 0.14 to 0.20. Moreover, the Type I error does not decrease as the sample size increases, indicating that the inflation is not only a finite-sample phenomenon. Instead, these results suggest that omitting the penalty on θ could lead to a fundamental violation of Type I error control, even asymptotically.

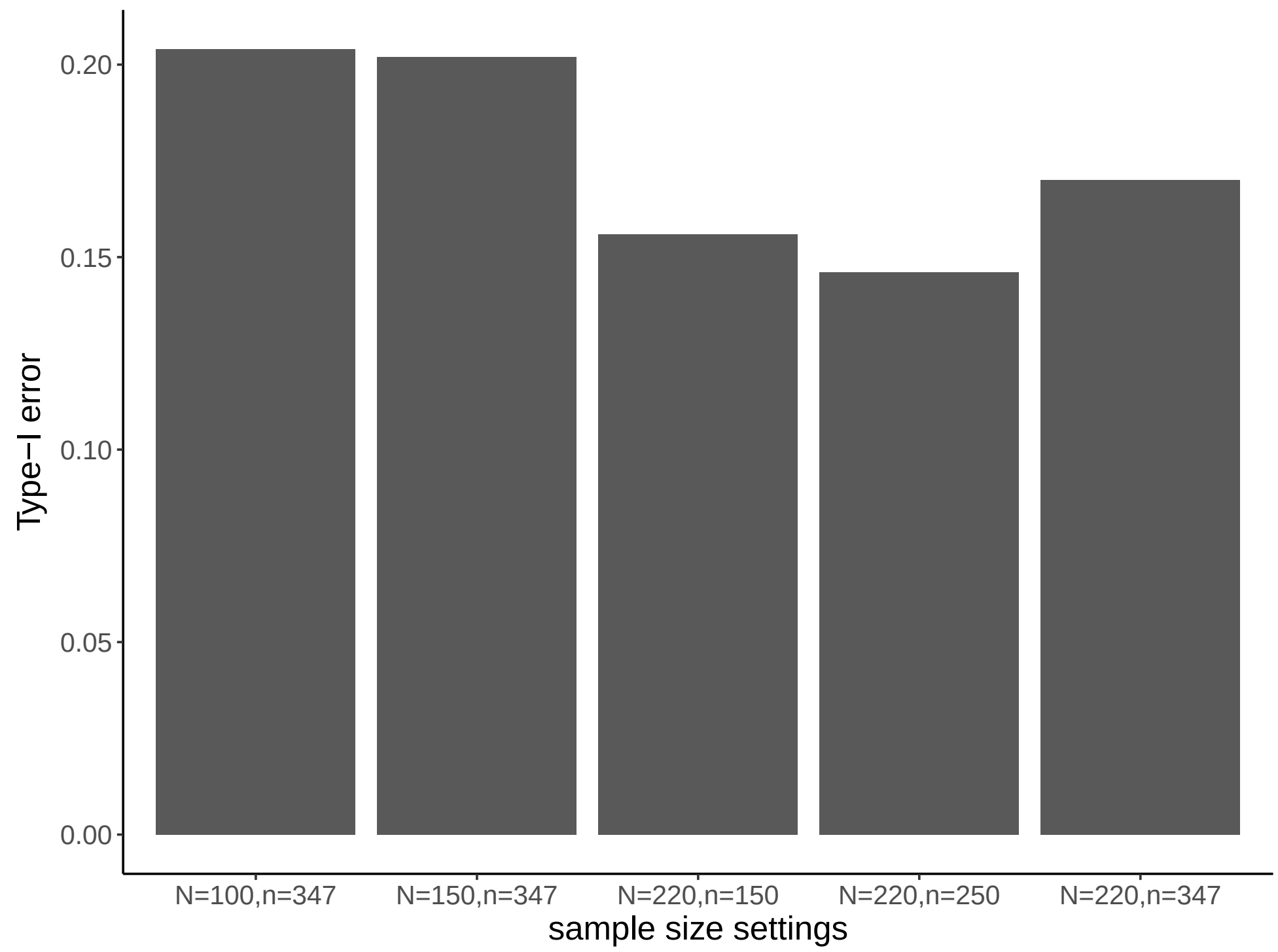

Figure S4: Type I error under different sample size settings if no penalty for θ in the stage 2 model when $\sigma_\delta = \sigma_\varepsilon = 4$.

1.4 Simulation results using existing debiasing method

Here, we evaluated the debiasing method of Zhang and Zhang (2014), one of the earliest approaches for inference in high-dimensional linear models. Specifically, we implemented the method using the `lasso.proj` function in the *R* package *hdi*. Because `lasso.proj` relies on computationally intensive nodewise regressions, applying it to all possible reference taxa is computationally prohibitive. Therefore, we considered a single reference taxon in the simulation study for illustration. All other simulation settings were identical to those in the main text (page 6), with $\sigma_\delta = \sigma_\varepsilon = 4$.

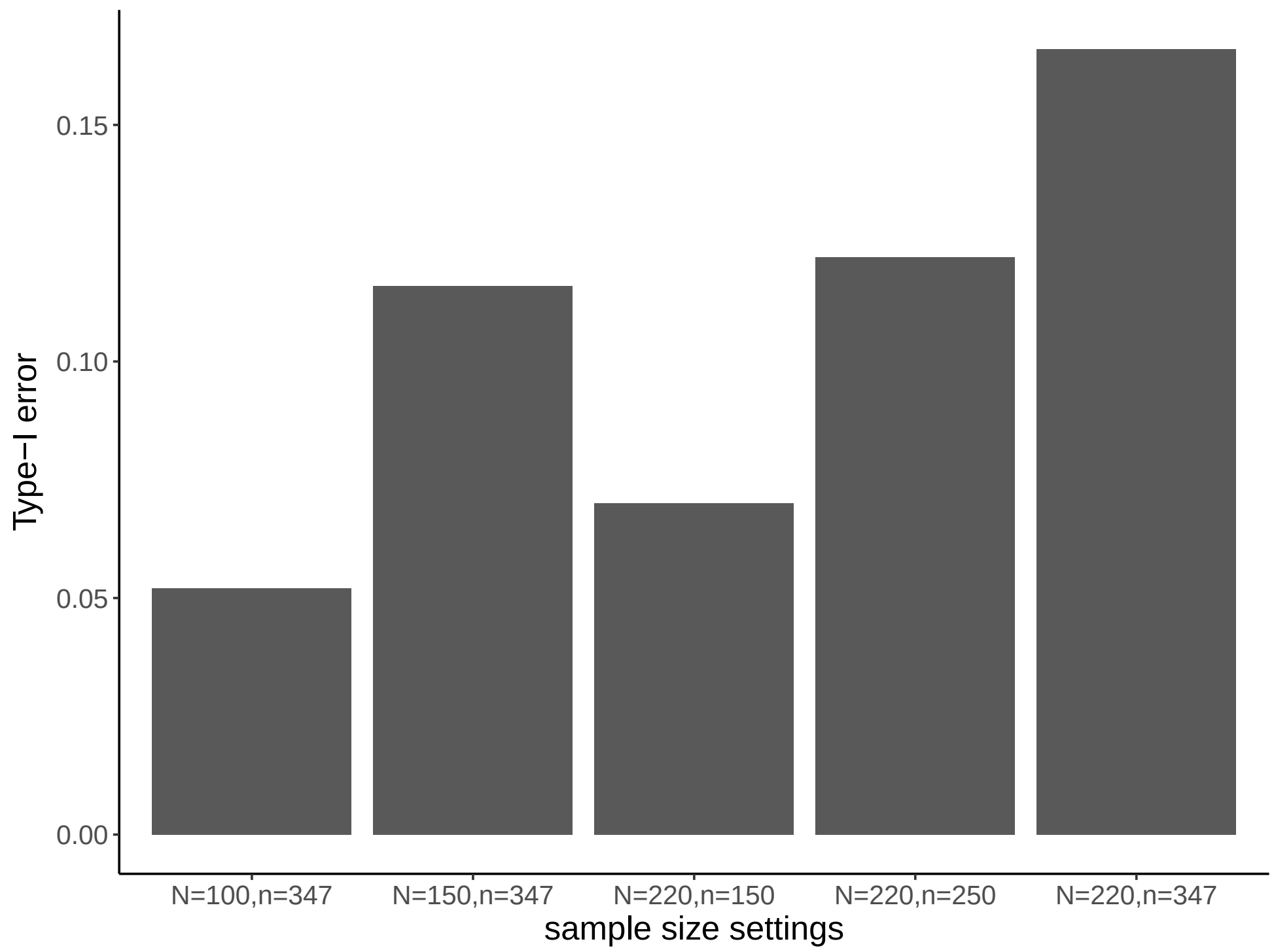

Figure S5: Type I error under different sample size settings if directly applying the debiasing Lasso method by R function `lasso.proj` when $\sigma_\delta = \sigma_\varepsilon = 4$.

Figure S5 summarizes the empirical Type I error rates. In our simulation setting, the `lasso.proj` procedure does not adequately control the Type I error for most sample size configurations. Moreover, the empirical Type I error tends to increase as either the external or target sample size increases.

2 Additional Real Data Analysis Results

2.1 Additional microbiome-metabolite-IBD pathway diagrams

Fig. S6 illustrates two diagrams for the microbiome-N1-Acetylspermidine-IBD and microbiome-Sphinganine-IBD pathway. Fig. S7 illustrates two diagrams for the microbiome-Nicothinic acid-IBD and microbiome-Lithocholic acid glycine conjugate-IBD pathway. Fig. S8 illustrates the diagram for the microbiome-4-Pyridoxic acid-IBD pathway.

2.2 Secondary Analysis

We conducted a secondary analysis restricted to the 17 shared metabolites using only the target dataset (FRAN, $n = 153$). Specifically, we considered the target model

$$y_i = m_i\theta^* + \mathbf{x}_i^\intercal \boldsymbol{\beta}^* + \epsilon_i, \quad i = 1, \ldots, n,$$

(A)

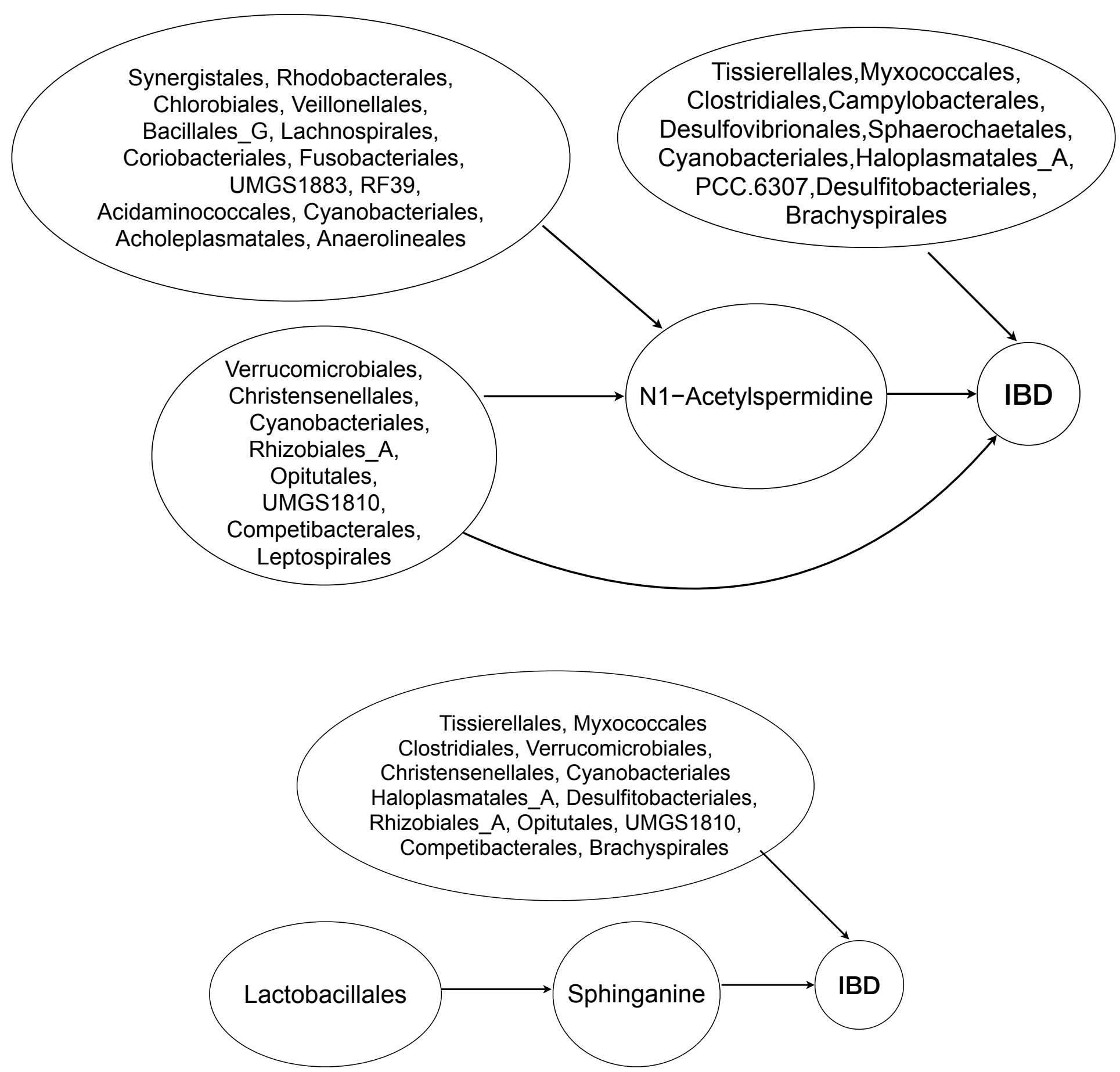

Figure S6: (A) Diagram of microbiome-N1-Acetylspermidine-IBD pathway: 33 microbial taxa were identified as participating in this pathway and are grouped into three functional categories. (B) Diagram of microbiome-Sphinganine-IBD pathway: 14 microbial taxa were identified as participating in this pathway and are grouped into three functional categories.

(A)

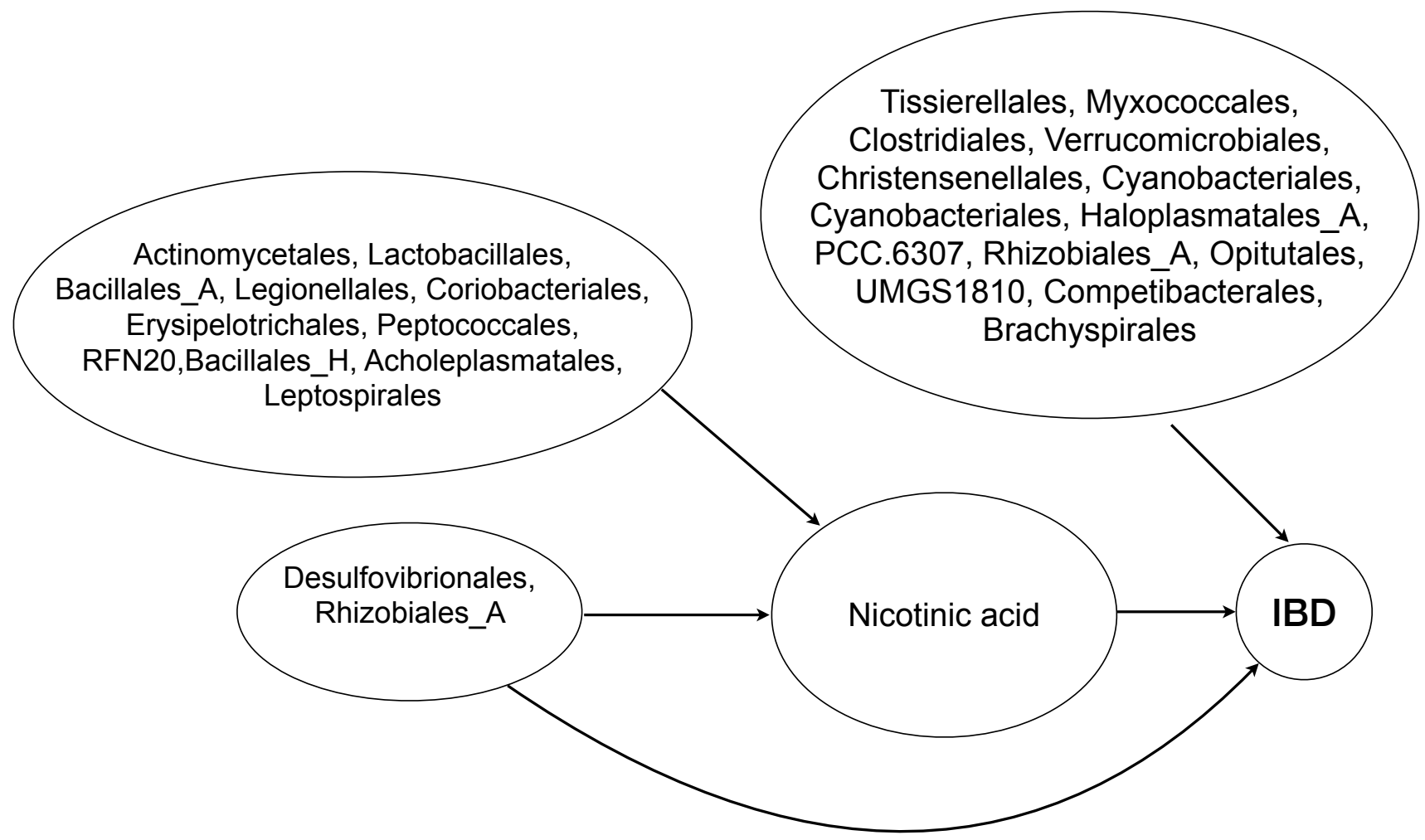

(B)

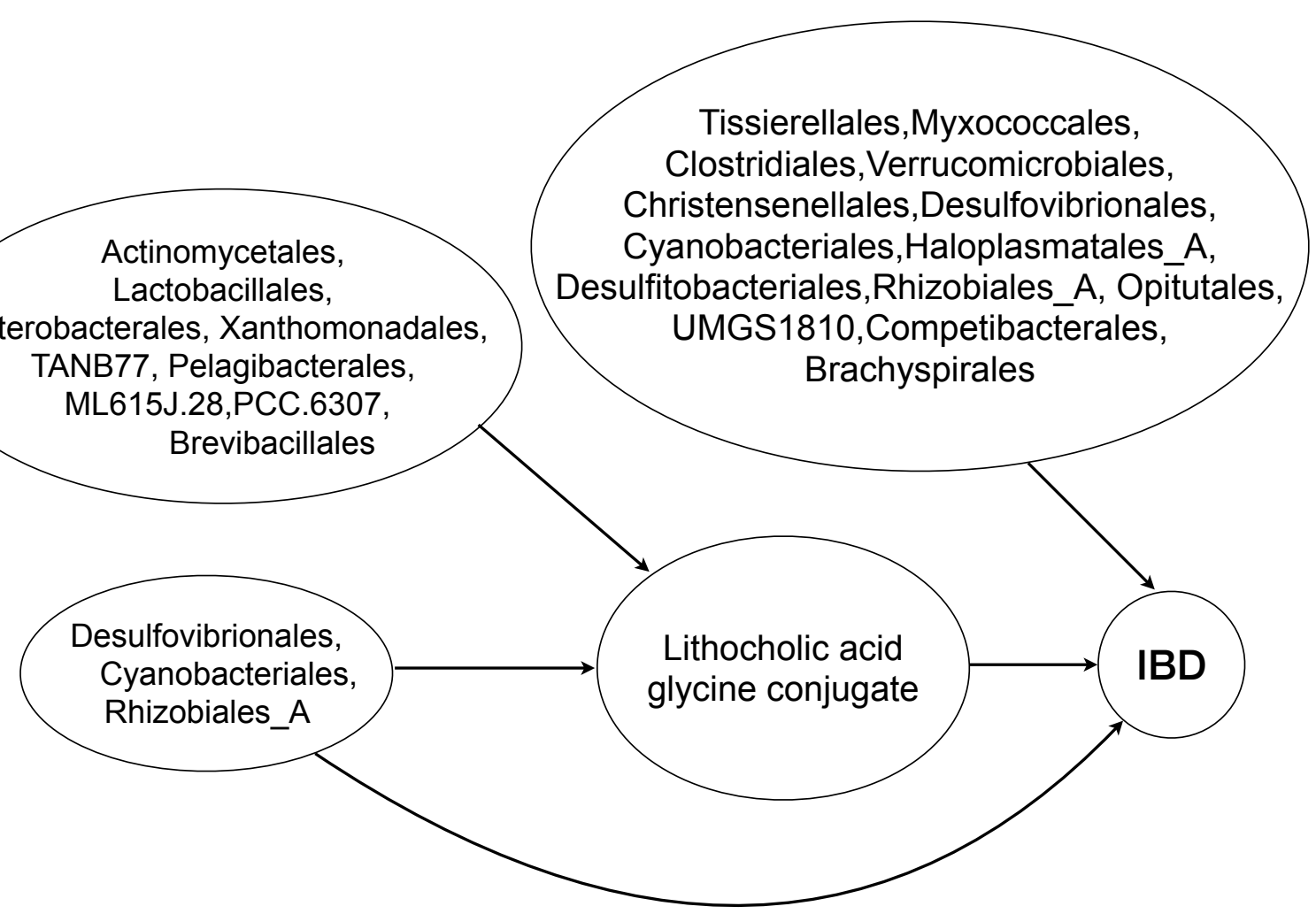

Figure S7: (A) Diagram of microbiome-Nicotinic acid-IBD pathway: 26 microbial taxa were identified as participating in this pathway and are grouped into three functional categories. (B) Diagram of microbiome-Sphinganine-IBD pathway: 26 microbial taxa were identified as participating in this pathway and are grouped into three functional categories.

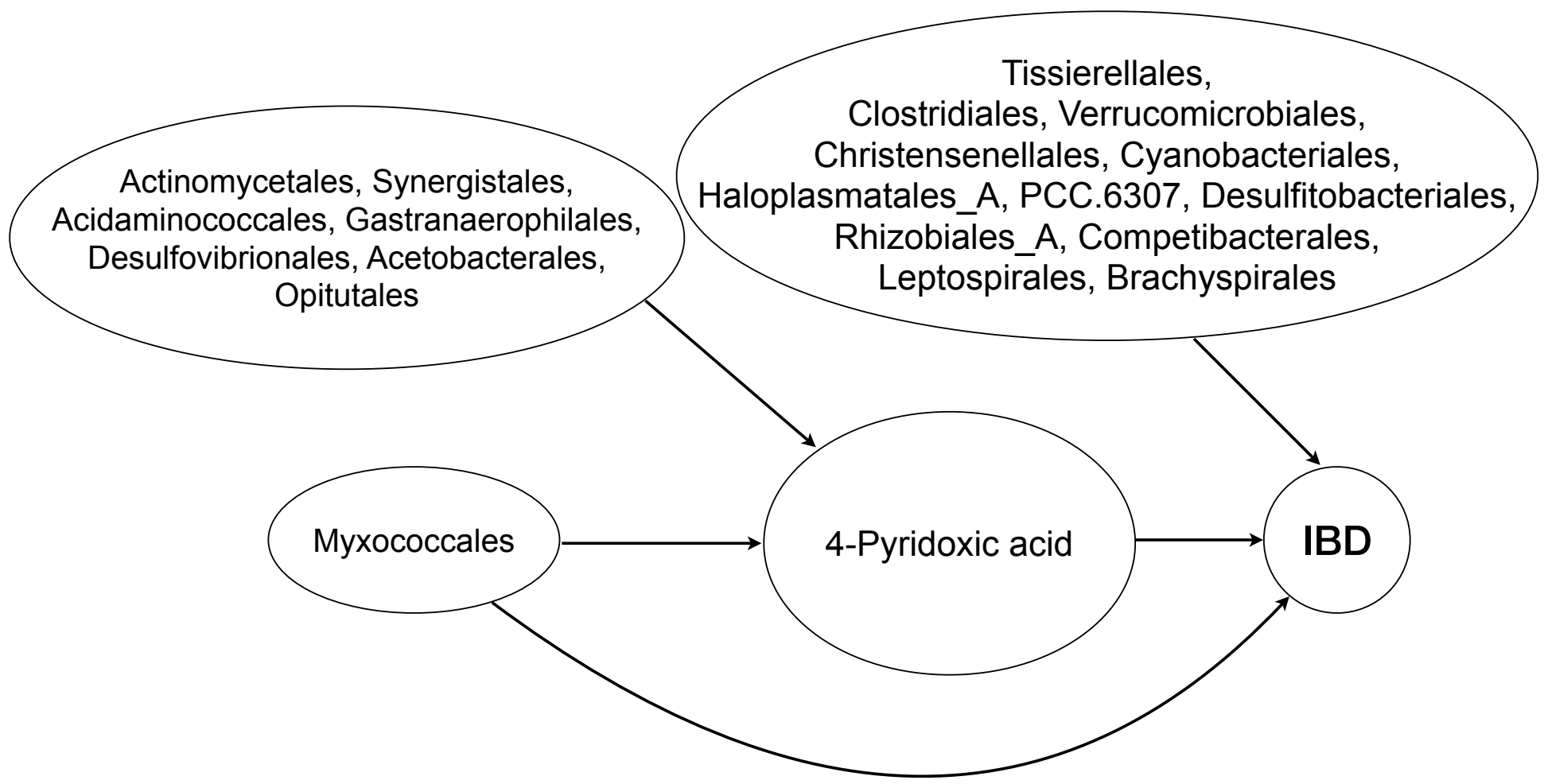

Figure S8: Diagram of microbiome-4-Pyridoxic acid-IBD pathway: 20 microbial taxa were identified as participating in this pathway and are grouped into three functional categories.

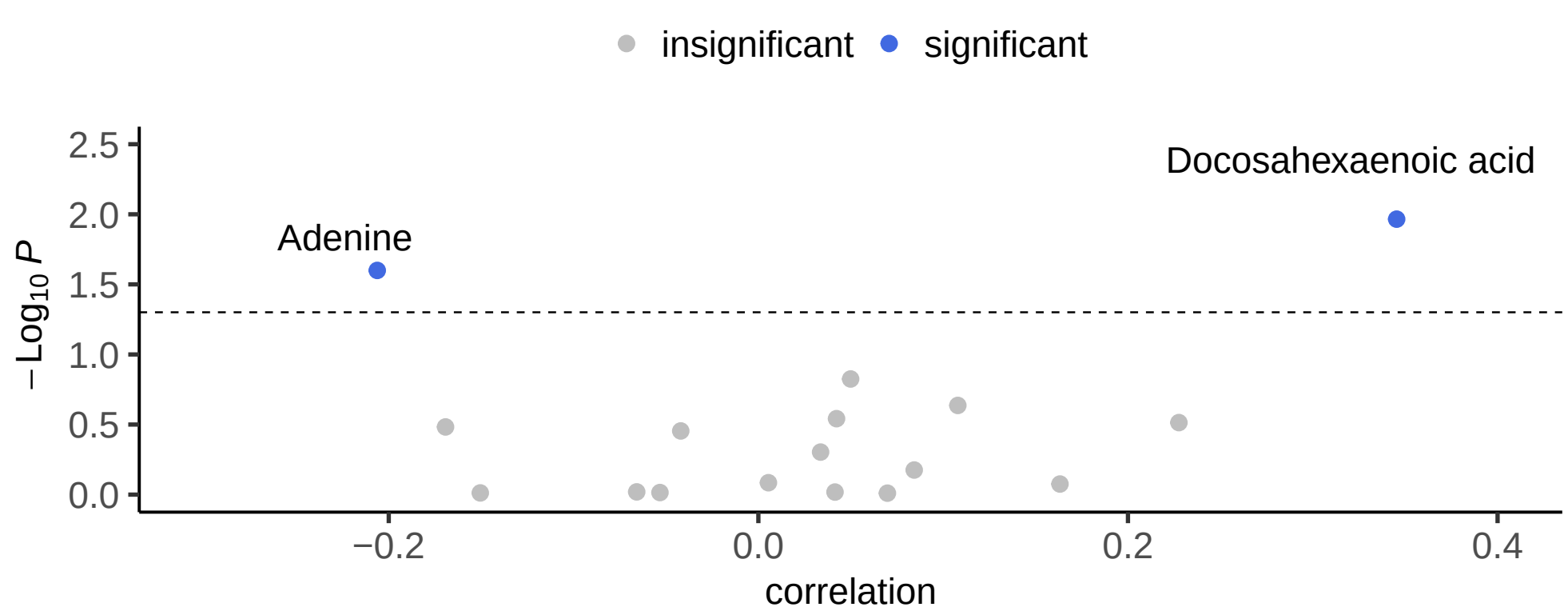

Figure S9: Volcano plot showing two IBD-associated metabolites identified by the target-only method using the observed 17 metabolites in the FRAN dataset. Blue dots indicate significant metabolites ($p < 0.05$), and grey dots indicate non-significant metabolites. The dashed line marks the nominal significance level of $-\log_{10}(0.05)$.

where m_i is the observed metabolite abundance in the FRAN dataset, and $\mathbf{x}_i$ is the ALR-transformed microbiome data using a specific reference microbe. We then obtained the penalized estimates

$$(\widehat{\theta}, \widehat{\boldsymbol{\beta}}) = \text{argmin}_{\theta,\boldsymbol{\beta}} \left\{ \frac{1}{2n} \sum_{i=1}^{n} \left(y_i - m_i\theta - \mathbf{x}_i^{\intercal}\boldsymbol{\beta} \right)^2 + \lambda_{\beta} \left(\|\boldsymbol{\beta}\|_1 + |\theta| \right) \right\}. \tag{9}$$

Using the identified support of $\widehat{\boldsymbol{\beta}}$, we applied the same regularized permutation test as described in the main text to obtain a p-value for testing $H_0 : \theta^* = 0$. Finally, following the idea of our robust multi-reference procedure, we repeated this analysis using each microbe as a reference and combined the resulting p-values via the Cauchy combination test.

We note that this target-only approach is methodologically different from the penalized two-stage least-squares approach described in the main text. In the target-only model, if unmeasured confounding exists (i.e., m_i and ϵ_i are correlated), the estimators in (9) may be biased. In contrast, our penalized two-stage least-squares approach uses the predicted metabolite $\widehat{m}_i$, obtained from an independent external dataset, which helps to mitigate this confounding bias.

As shown in Fig. S9, only two metabolite, docosahexaenoic acid and adenine, reached marginal significance. After FDR adjustment, it was no longer significant, consistent with the results reported in the main paper. The two significant shared metabolites identified by our integrative approach in the main text were not significant in this target-only analysis. This difference may reflect the methodological distinction between the two approaches described previously, namely whether unmeasured confounding between the metabolites and the outcome can be accounted for.

2.3 Sensitivity analysis of different pseudo abundance values used in the microbiome data

To assess the sensitivity of our results to the choice of pseudo-abundance, we conducted additional analyses using two alternative values: one-half of the minimum observed nonzero relative abundance (3.64×10^{-8}) and 10^{-9}.

We then applied the proposed framework separately to each transformed dataset obtained by adding one of the three pseudo-abundances. For each analysis, we obtained a p-value for the 76 metabolites of interest in iHMP dataset. We then compared the resulting significance levels by constructing pairwise scatter plots of the corresponding $-\log_{10}(p)$ values and calculating the Pearson correlation coefficient for each pair, as shown in Fig. S10. We further compared the metabolites identified as significant using a nominal significance level of 0.05. The overlap of significant metabolites across the three pseudo-abundance choices is shown in the Venn diagram in Fig. S11.

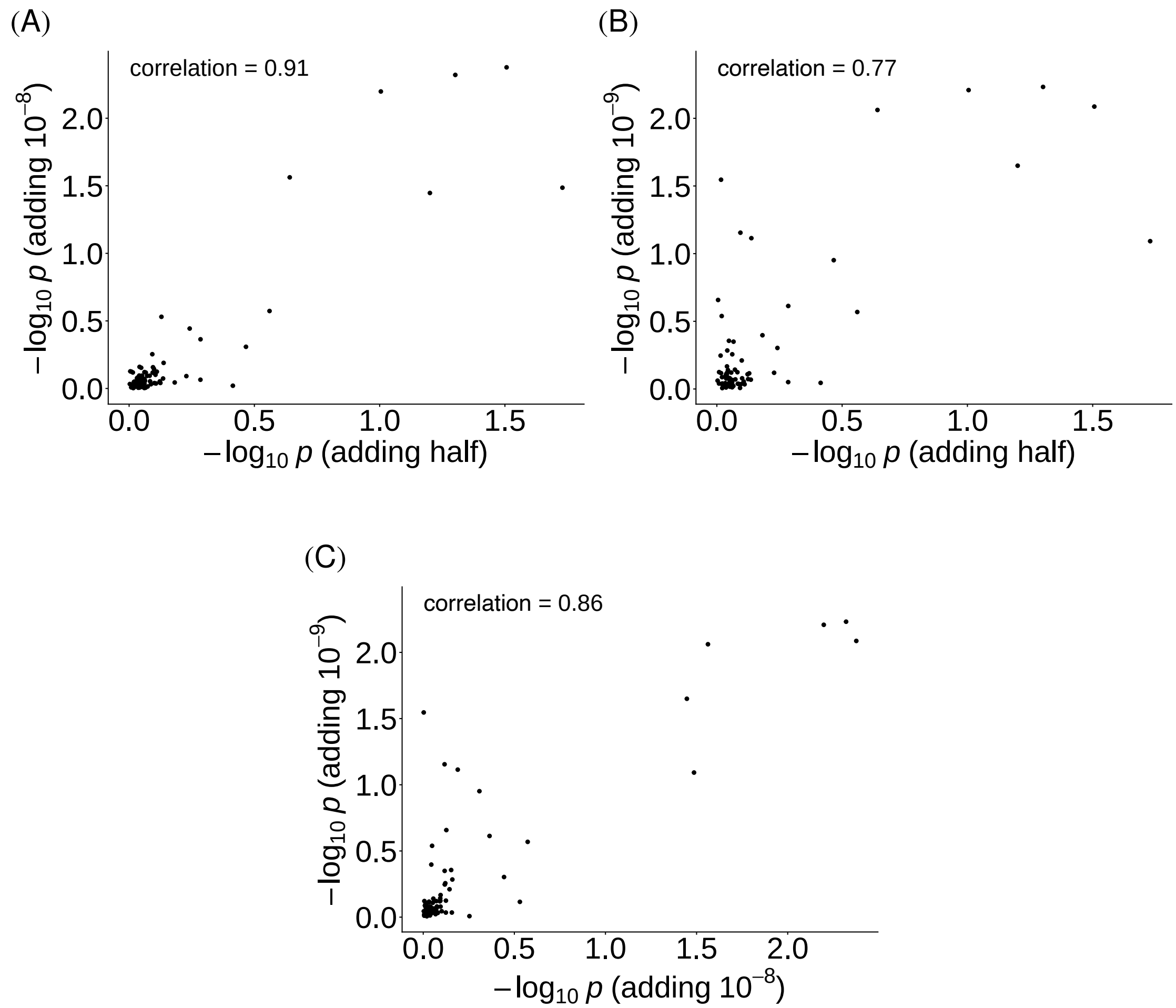

Figure S10: (A) Scatter plot of the $-\log_{10}$-transformed p-values for metabolites from iHMP-only and iHMP-share dataset when adding pseudo half of the minimum observed relative abundance (3.64×10^{-8}) versus 10^{-8} for preprocessing microbiome data. (B) Scatter plot of the $-\log_{10}$-transformed p-values for metabolites from iHMP-only and iHMP-share dataset when adding pseudo half of the minimum observed relative abundance (3.64×10^{-8}) versus 10^{-9} for preprocessing microbiome data. (C) Scatter plot of the $-\log_{10}$-transformed p-values for metabolites from iHMP-only and iHMP-share dataset when adding pseudo relative abundance 10^{-8} versus 10^{-9} for preprocessing microbiome data.

3 Theoretical Result and Technical Proofs

We present some theoretical results for the estimators obtained from the penalized two-stage least squares procedure. We start with the following assumptions:

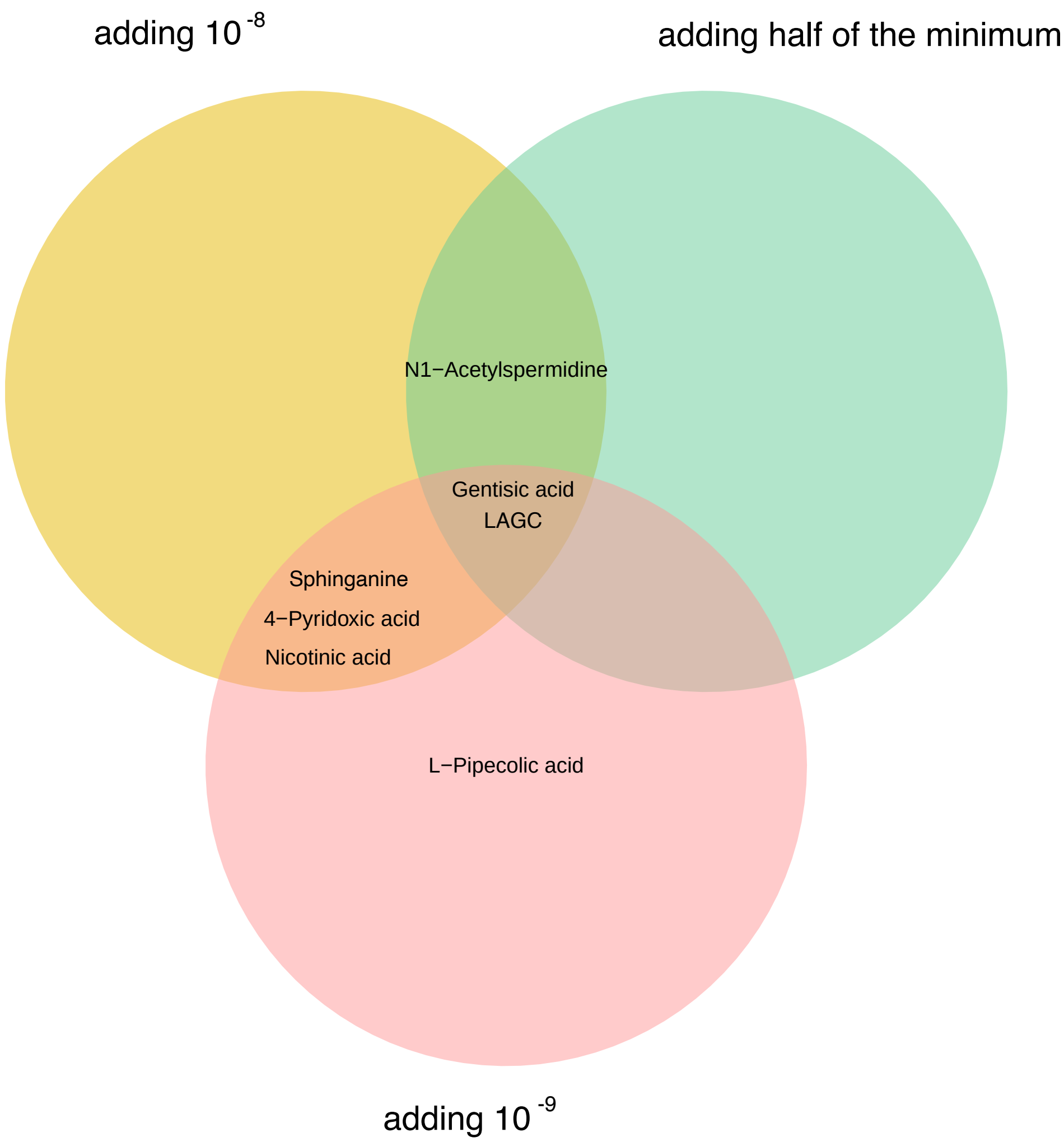

Figure S11: Venn diagram of significant metabolites identified from iHMP-only and iHMP-share under different ways of handling zeros. The LAGC represents metabolite lithocholic acid glycine conjugate.

Assumption 1. *We assume that $\widetilde{m}_j$ and $\widetilde{\mathbf{x}}_j$ follows the same microbiome-metabolome model in model (3).*

Assumption 2. *The true model (1) and (2) satisfy the plurality condition such that* $|\mathcal{G}_2| > \max_{c\neq 0} |l \in \mathcal{G}_1 \cup \mathcal{G}_2 : \beta_l^0/\gamma_l^0 = c|$.

Assumption 3. *(a) For the ALR transformed external data, there exists a constant $\phi_{0\gamma}^2$ such that $\|\widetilde{\mathbf{X}}\Delta\|_2^2 \geq N\phi_{0\gamma}^2\|\Delta_{S_0}\|_2^2$ for all $\Delta \in \mathbb{R}^{p-1}$ satisfying $\|\Delta_{S_0^c}\|_1 \leqslant 3\|\Delta_{S_0}\|_1$, where $S_0 := \{j : \gamma_j^* \neq 0\}$ and $\widetilde{\mathbf{X}} = (\widetilde{\mathbf{x}}_1, \ldots, \widetilde{\mathbf{x}}_n)^\intercal$.*

(b) For the ALR transformed target data, there exists a constant $\phi_{0_\beta}^2$ such that $\|\mathbf{X}_M\Delta\|_2^2 \geq n\phi_{0_\beta}^2\|\Delta_{S_0'}\|_2^2$ for all $\Delta \in \mathbb{R}^p$ satisfying $\|\Delta_{S_0'^c}\|_1 \leqslant 3\|\Delta_{S_0'}\|_1$, where $S_0' := \{1\} \cup \{j+1 : \beta_j^* \neq 0\}$, and $\mathbf{X}_M$ is an $n \times p$ matrix with the i-th row being $(\mathbf{x}_i^\intercal\boldsymbol{\gamma}^*, \mathbf{x}_i^\intercal)$ for $i = 1, \ldots, n$.

Remark 3.1. *Assumption 1 ensures the informativeness of the external data. Assumption 2 is required for θ^* to be identifiable; see the proof in (Guo et al., 2018) . It states that $\mathcal{G}_2$ has the largest number of microbes that provide the same estimation of θ^*, implying $|\mathcal{G}_2| \geq 2$. Assumptions 3(a) and 3(b) are the restricted eigenvalue conditions (REC) for (3) and (4), respectively; see further discussion of REC in (Bickel et al., 2009).*

Assumption 4. *$min_{j:\beta_j^*\neq 0} |\beta_j^*| > 8\lambda_\beta s_{0_\beta}/\phi_{0_\beta}^2$, where $s_{0_\beta} = \|\boldsymbol{\beta}^*\|_0$.*

The next result establishes the variable selection consistency.

Theorem 1. *The thresholded Lasso estimators $\widehat{\beta}_{\text{thres},j} = \widehat{\beta}_j \mathbb{1}(|\widehat{\beta}_j| \geq C_2')$ where $\widehat{\beta}_j$ is defined in (6) with $C_2' \geq 4\lambda_\beta s_{0_\beta}/\phi_{0_\beta}^2$ satisfy $P(\text{sign}(\widehat{\boldsymbol{\beta}}_{\text{thres}}) = \text{sign}(\boldsymbol{\beta}^*)) \to 1$ as $N, n \to \infty$, where sign $(\cdot)$ is the sign function that maps a positive entry to 1, a negative entry to -1, and zero to zero.*

Assuming $\min_{j:\gamma_j^*\neq 0} |\gamma_j^*| > 8\lambda_\gamma s_{0_\gamma}/\phi_{0_\gamma}^2$, where $s_{0_\gamma} = \|\boldsymbol{\gamma}^*\|_0$, we define $\widehat{\gamma}_{\text{thres},j} = \widehat{\gamma}_j \mathbb{1}(|\widehat{\gamma}_j| \geq C_2)$ with $C_2 \geq 4\lambda_\gamma s_{0_\gamma}/\phi_{0_\gamma}^2$. Like Theorem 1, $\widehat{\boldsymbol{\gamma}}_{\text{thres}}$ can consistently identify the support of $\boldsymbol{\gamma}^*$.

Therefore, we can estimate $\widehat{\mathcal{G}}_1$, $\widehat{\mathcal{G}}_2$, $\widehat{\mathcal{G}}_3$, $\widehat{\mathcal{G}}_4$ based on the support of thresholded estimators $\widehat{\boldsymbol{\gamma}}_{\text{thres}}$ and $\widehat{\boldsymbol{\beta}}_{\text{thres}}$.

For ease of notation, we let $a \vee b$ represents $\max\{a, b\}$ and $a \wedge b$ denotes $\min\{a, b\}$.

3.1 Proof of Theorem 1

Proof. We start from proving the estimation consistency, then the variable selection consistency.

3.1.1 Proof of the estimation consistency

Proposition 3.1. *If Assumptions 1-3 hold, and $\lambda_\gamma = O(\sqrt{\log p/N})$, then $\|\widehat{\boldsymbol{\gamma}} - \boldsymbol{\gamma}^*\|_1 = O_p(\lambda_\gamma s_{0_\gamma})$, where $s_{0_\gamma} = \|\boldsymbol{\gamma}^*\|_0$. Similarly, we have $\|\widehat{\boldsymbol{\beta}} - \boldsymbol{\beta}^*\|_1 = O_p(\lambda_\beta s_{0_\beta})$ for $\lambda_\beta = O(\lambda_\gamma s_{0_\gamma}^2 \vee \sqrt{\log p/n})$. If in addition, $s_{0_\beta} = o(\sqrt{N/(s_{0_\gamma}^2 \log p)} \wedge \sqrt{n/\log p})$, we have $\|(\widehat{\theta}, \widehat{\boldsymbol{\beta}}) - (\theta^*, \boldsymbol{\beta}^*)\|_1 \xrightarrow{p} 0$.*

We start with the stage 1 estimation for $\widehat{\boldsymbol{\gamma}}$, by the classic Lasso and REC Assumption 3(a), we have $\|\widehat{\boldsymbol{\gamma}} - \boldsymbol{\gamma}^*\|_1 = O_p(\lambda_\gamma s_{0_\gamma})$ (Bühlmann and Van De Geer, 2011).

By the definition of $\widehat{\theta}$ and $\widehat{\boldsymbol{\beta}}$:

$$\left(\widehat{\theta}, \widehat{\boldsymbol{\beta}}\right) = \underset{\theta, \boldsymbol{\beta}^*}{\operatorname{argmin}} \sum_{i=1}^{n} \frac{\left(y_i - \widehat{m}_i \theta - \mathbf{x}_i^\intercal \boldsymbol{\beta}\right)^2}{2n} + \lambda_\beta \left\{\|\boldsymbol{\beta}\|_1 + |\theta|\right\},$$

we have

$$\frac{1}{n}\|\mathbf{y} - \widehat{\theta}\widehat{\mathbf{m}} - \mathbf{X}\widehat{\boldsymbol{\beta}}\|_2^2 + \lambda_\beta \|(\widehat{\theta}, \widehat{\boldsymbol{\beta}})\| \leqslant \frac{1}{n}\|\mathbf{y} - \theta^*\widehat{\mathbf{m}} - \mathbf{X}\boldsymbol{\beta}^*\|_2^2 + \lambda_\beta \|(\theta^*, \boldsymbol{\beta}^*)\|_1.$$

Plugging in $\mathbf{y} = \theta^*\mathbf{m} + \mathbf{X}\boldsymbol{\beta}^* + \boldsymbol{\varepsilon}$ and $\mathbf{m} = \mathbf{X}\boldsymbol{\gamma}^* + \boldsymbol{\delta}$, we have

$$\begin{aligned} &\frac{1}{n}\|\theta^*\mathbf{X}\boldsymbol{\gamma}^* + \mathbf{X}\boldsymbol{\beta}^* + \boldsymbol{\delta}\theta^* + \boldsymbol{\varepsilon} - \widehat{\mathbf{m}}\widehat{\theta} - \mathbf{X}\widehat{\boldsymbol{\beta}}\|_2^2 + \lambda\|(\widehat{\theta}, \widehat{\boldsymbol{\beta}})\| \\ &\leqslant \frac{1}{n}\|(\mathbf{X}\boldsymbol{\gamma}^* - \widehat{\mathbf{m}})\theta^* + \boldsymbol{\delta}\theta^* + \boldsymbol{\varepsilon}\|_2^2 + \lambda_\beta\|(\theta^*, \boldsymbol{\beta}^*)\|. \end{aligned}$$

Reorganizing the above inequality, we have

$$\begin{aligned} &\frac{1}{n}\|(\mathbf{X}\boldsymbol{\gamma}^* - \widehat{\mathbf{m}})\theta^* + \boldsymbol{\delta}\theta^* + \boldsymbol{\varepsilon}\|_2^2 + \lambda_\beta\|(\theta^*, \boldsymbol{\beta}^*)\|_1 \\ \geq &\frac{1}{n}\|\theta^*\mathbf{X}\boldsymbol{\gamma}^* + \mathbf{X}\boldsymbol{\beta}^* + \boldsymbol{\delta}\theta^* + \boldsymbol{\varepsilon} - \widehat{\mathbf{m}}\widehat{\theta} - \mathbf{X}\widehat{\boldsymbol{\beta}}\|_2^2 + \lambda_\beta\|(\widehat{\theta}, \widehat{\boldsymbol{\beta}})\|_1 \\ = &\frac{1}{n}\|\widehat{\mathbf{m}}(\theta^* - \widehat{\theta}) + \mathbf{X}(\boldsymbol{\beta}^* - \widehat{\boldsymbol{\beta}}) + (\mathbf{X}\boldsymbol{\gamma}^* - \widehat{\mathbf{m}})\theta^* + \boldsymbol{\delta}\theta^* + \boldsymbol{\varepsilon}\|_2^2 + \lambda_\beta\|(\widehat{\theta}, \widehat{\boldsymbol{\beta}})\|_1. \end{aligned}$$

Thus, we get

$$\begin{aligned} \frac{1}{n}\|\widehat{\mathbf{m}}(\theta^* - \widehat{\theta}) + \mathbf{X}(\boldsymbol{\beta}^* - \widehat{\boldsymbol{\beta}})\|_2^2 \leqslant &\frac{2}{n}(\widehat{\mathbf{m}}(\widehat{\theta} - \theta^*) + \mathbf{X}(\widehat{\boldsymbol{\beta}} - \boldsymbol{\beta}^*))^\intercal((\mathbf{X}\boldsymbol{\gamma}^* - \widehat{\mathbf{m}})\theta^* + \boldsymbol{\delta}\theta^* + \boldsymbol{\varepsilon}) \\ &+ \lambda_\beta\|(\theta^*, \boldsymbol{\beta}^*)\|_1 - \lambda_\beta\|(\widehat{\theta}, \widehat{\boldsymbol{\beta}})\|_1 \\ = &\underbrace{\frac{2}{n}(\widehat{\theta} - \theta^*)\widehat{\mathbf{m}}^\intercal(\mathbf{X}\boldsymbol{\gamma}^* - \widehat{\mathbf{m}})\theta^*}_{\text{(i)}} + \underbrace{\frac{2}{n}(\widehat{\theta} - \theta^*)\widehat{\mathbf{m}}^\intercal(\boldsymbol{\delta}\theta^* + \boldsymbol{\varepsilon})}_{\text{(ii)}} \\ &+ \underbrace{\frac{2}{n}\theta^*(\widehat{\boldsymbol{\beta}} - \boldsymbol{\beta}^*)^\intercal\mathbf{X}^\intercal(\mathbf{X}\boldsymbol{\gamma}^* - \widehat{\mathbf{m}})}_{\text{(iii)}} + \underbrace{\frac{2}{n}(\widehat{\boldsymbol{\beta}} - \boldsymbol{\beta}^*)^\intercal\mathbf{X}^\intercal(\boldsymbol{\delta}\theta^* + \boldsymbol{\varepsilon})}_{\text{(iv)}} \\ &+ \underbrace{\lambda_\beta\|(\theta^*, \boldsymbol{\beta}^*)\|_1 - \lambda_\beta\|(\widehat{\theta}, \widehat{\boldsymbol{\beta}})\|_1}_{\text{(v)}} \end{aligned} \tag{10}$$

Denote the event $S := \{\max_{1<j<p}|\mathbf{x}_{\cdot j}^\intercal(\boldsymbol{\delta}\theta^* + \boldsymbol{\varepsilon})| \quad \vee \quad |(\mathbf{X}\boldsymbol{\gamma}^*)^\intercal(\boldsymbol{\delta}\theta^* + \boldsymbol{\varepsilon})| \leq n\lambda_{01}/2\}$, where $\mathbf{x}_{\cdot j}$ is the j-th column of $\mathbf{X}$. Hereafter, the argument will be based on the event S. We know that $\boldsymbol{\delta}\theta^* + \boldsymbol{\varepsilon}$ follows a normal distribution since $(\delta_i, \varepsilon_i)$ is a bivariate normal random vector. Thus, given $\mathbf{x}_{\cdot j}$, we have $\mathbf{x}_{\cdot j}^\intercal(\boldsymbol{\delta}\theta^* + \boldsymbol{\varepsilon})$ follows a normal distribution and $(\mathbf{X}\boldsymbol{\gamma}^*)^\intercal(\boldsymbol{\delta}\theta^* + \boldsymbol{\varepsilon}) =$

$\sum_j \gamma_j \mathbf{x}_{\cdot j}^{\intercal}(\boldsymbol{\delta}\theta^* + \boldsymbol{\varepsilon}) = O_p(s_{0_\gamma} \mathbf{x}_{\cdot j}^{\intercal}(\boldsymbol{\delta}\theta^* + \boldsymbol{\varepsilon}))$. So the event S holds with high probability if $\lambda_{01} \asymp s_{0_\gamma}\sqrt{\log p/n}$.

For term (ii), since $\boldsymbol{\varepsilon}$ is not independent of $\widehat{\mathbf{m}}$, we do the following decomposition

$$\begin{aligned}\frac{1}{n}\widehat{\mathbf{m}}^{\intercal}(\boldsymbol{\delta}\theta^* + \boldsymbol{\varepsilon}) &= \frac{1}{n}(\mathbf{X}(\widehat{\boldsymbol{\gamma}} - \boldsymbol{\gamma}^*))^{\intercal}(\boldsymbol{\delta}\theta^* + \boldsymbol{\varepsilon}) + \frac{1}{n}(\mathbf{X}\boldsymbol{\gamma}^*)^{\intercal}(\boldsymbol{\delta}\theta^* + \boldsymbol{\varepsilon}) \\ &\le \frac{1}{n}\|\widehat{\boldsymbol{\gamma}} - \boldsymbol{\gamma}^*\|_1 \cdot \|\mathbf{X}^{\intercal}(\boldsymbol{\delta}\theta^* + \boldsymbol{\varepsilon})\|_\infty + \frac{1}{n}(\mathbf{X}\boldsymbol{\gamma}^*)^{\intercal}(\boldsymbol{\delta}\theta^* + \boldsymbol{\varepsilon})\end{aligned}$$

Given S, for terms (ii) and (iv), we have $\frac{1}{n}|\widehat{\mathbf{m}}^{\intercal}(\boldsymbol{\delta}\theta^* + \boldsymbol{\varepsilon})| \leqslant \frac{\lambda_{01}}{2}\|\widehat{\boldsymbol{\gamma}} - \boldsymbol{\gamma}^*\|_1 + \frac{\lambda_{01}}{2}$ and $\frac{2}{n}|(\widehat{\boldsymbol{\beta}} - \boldsymbol{\beta}^*)^{\intercal}\mathbf{X}^{\intercal}(\boldsymbol{\delta}\theta^* + \boldsymbol{\varepsilon})| \leqslant \lambda_{01}\|\boldsymbol{\beta}^* - \widehat{\boldsymbol{\beta}}\|_1$.

For the term (i), we have

$$\begin{aligned}\frac{1}{n}(\widehat{\theta} - \theta^*)\widehat{\mathbf{m}}^{\intercal}(\mathbf{X}\boldsymbol{\gamma}^* - \widehat{\mathbf{m}})\theta^* &= (\widehat{\theta} - \theta^*)\theta^* \cdot \widehat{\boldsymbol{\gamma}}^{\intercal}\widehat{\boldsymbol{\Sigma}}(\widehat{\boldsymbol{\gamma}} - \boldsymbol{\gamma}^*) \\ &\leqslant |\widehat{\theta} - \theta^*| \cdot |\theta^*| \cdot \|\widehat{\boldsymbol{\gamma}} - \boldsymbol{\gamma}^*\|_1 \cdot \|\widehat{\boldsymbol{\Sigma}}\widehat{\boldsymbol{\gamma}}\|_\infty \\ &= O(|\widehat{\theta} - \theta^*| \cdot |\theta^*| \cdot \|\widehat{\boldsymbol{\gamma}} - \boldsymbol{\gamma}^*\|_1 \cdot \|\widehat{\boldsymbol{\gamma}}\|_1) \\ &= O_p(|\widehat{\theta} - \theta^*| \cdot |\theta^*| \cdot \|\widehat{\boldsymbol{\gamma}} - \boldsymbol{\gamma}^*\|_1 \cdot s_{0_\gamma}), \qquad (11)\end{aligned}$$

where $\widehat{\boldsymbol{\Sigma}} = \mathbf{X}^{\intercal}\mathbf{X}/n$ and the last equality holds since $\|\widehat{\boldsymbol{\gamma}}\|_1 = O_p(s_{0_\gamma})$ from Zhang and Huang (2008).

For term (iii), we have

$$\begin{aligned}\frac{1}{n}(\widehat{\boldsymbol{\beta}} - \boldsymbol{\beta}^*)^{\intercal}\mathbf{X}^{\intercal}(\mathbf{X}\boldsymbol{\gamma}^* - \widehat{\mathbf{m}}) &= (\widehat{\boldsymbol{\beta}} - \boldsymbol{\beta}^*)^{\intercal}\widehat{\boldsymbol{\Sigma}}(\boldsymbol{\gamma}^* - \widehat{\boldsymbol{\gamma}}) \\ &\leqslant \|(\widehat{\boldsymbol{\beta}} - \boldsymbol{\beta}^*)^{\intercal}\widehat{\boldsymbol{\Sigma}}\|_\infty \|\widehat{\boldsymbol{\gamma}} - \boldsymbol{\gamma}^*\|_1 \\ &= O(\|\boldsymbol{\beta}^* - \widehat{\boldsymbol{\beta}}\|_1 \cdot \|\widehat{\boldsymbol{\gamma}} - \boldsymbol{\gamma}^*\|_1).\end{aligned}$$

For term (v), we have $\lambda_\beta\|(\theta^*, \boldsymbol{\beta}^*)\|_1 - \lambda_\beta\|(\widehat{\theta}, \widehat{\boldsymbol{\beta}})\|_1 \le \lambda_\beta\|\widehat{\boldsymbol{\beta}} - \boldsymbol{\beta}^*\|_1 + \|\widehat{\theta} - \theta^*\|_1$.

Combining the inequalities for terms (i) to (v), together with the fact that $\|\widehat{\boldsymbol{\gamma}} - \boldsymbol{\gamma}^*\|_1 = O_p(\lambda_\gamma s_{0_\gamma})$ with $\lambda_\gamma \asymp \sqrt{\log p/N}$, we have

$$\frac{1}{n}\|\widehat{\mathbf{m}}(\theta^* - \widehat{\theta}) + \mathbf{X}(\boldsymbol{\beta}^* - \widehat{\boldsymbol{\beta}})\|_2^2 = O_p(\lambda_\beta|\theta^* - \widehat{\theta}| + \lambda_\beta\|\boldsymbol{\beta}^* - \widehat{\boldsymbol{\beta}}\|_1)$$

for the turning parameter $\lambda_\beta = O(\lambda_\gamma s_{0_\gamma}^2)$, which based on (11).

To complete the proof, we need to show the following :

$$\|(\theta^* - \widehat{\theta}, \boldsymbol{\beta}^*_{S_0'} - \widehat{\boldsymbol{\beta}}_{S_0'})\|_1^2 = O_p(\frac{1}{n}\|\widehat{\mathbf{m}}(\theta^* - \widehat{\theta}) + \mathbf{X}(\boldsymbol{\beta}^* - \widehat{\boldsymbol{\beta}})\|_2^2). \qquad (12)$$

In the RHS, we have

$$
\begin{aligned}
&\frac{1}{n}\|\widehat{\mathbf{m}}(\theta^* - \widehat{\theta}) + \mathbf{X}(\boldsymbol{\beta}^* - \widehat{\boldsymbol{\beta}})\|_2^2 \\
=&\frac{1}{n}\|\mathbf{X}\widehat{\boldsymbol{\gamma}}(\theta^* - \widehat{\theta}) + \mathbf{X}(\boldsymbol{\beta}^* - \widehat{\boldsymbol{\beta}})\|_2^2 \\
=&\frac{1}{n}\|\mathbf{X}\boldsymbol{\gamma}^*(\theta^* - \widehat{\theta}) + \mathbf{X}(\boldsymbol{\beta}^* - \widehat{\boldsymbol{\beta}}) + (\mathbf{X}\widehat{\boldsymbol{\gamma}} - \mathbf{X}\boldsymbol{\gamma}^*)(\theta^* - \widehat{\theta})\|_2^2 \\
=&\frac{1}{n}\|\mathbf{X}\boldsymbol{\gamma}^*(\theta^* - \widehat{\theta}) + \mathbf{X}(\boldsymbol{\beta}^* - \widehat{\boldsymbol{\beta}})\|_2^2 + \frac{2}{n}(\theta^* - \widehat{\theta})(\mathbf{X}\widehat{\boldsymbol{\gamma}} - \mathbf{X}\boldsymbol{\gamma}^*)^\intercal\mathbf{X}(\boldsymbol{\beta}^* - \widehat{\boldsymbol{\beta}}) \\
&+ \frac{2}{n}(\theta^* - \widehat{\theta})(\mathbf{X}\widehat{\boldsymbol{\gamma}} - \mathbf{X}\boldsymbol{\gamma}^*)^\intercal\mathbf{X}\boldsymbol{\gamma}^*(\theta^* - \widehat{\theta}) + \frac{1}{n}\|(\mathbf{X}\widehat{\boldsymbol{\gamma}} - \mathbf{X}\boldsymbol{\gamma}^*)(\theta^* - \widehat{\theta})\|_2^2
\end{aligned}
$$

If $s_{0_\gamma} = o(1/\lambda_\gamma)$, then $\|\widehat{\boldsymbol{\gamma}} - \boldsymbol{\gamma}^*\|_1 = o_p(1)$, accordingly we have

$$
\begin{aligned}
&(\theta^* - \widehat{\theta})(\mathbf{X}\widehat{\boldsymbol{\gamma}} - \mathbf{X}\boldsymbol{\gamma}^*)^\intercal\mathbf{X}(\boldsymbol{\beta}^* - \widehat{\boldsymbol{\beta}}) \\
&= o_p((\theta^* - \widehat{\theta})(\boldsymbol{\gamma}^*)^\intercal\mathbf{X}^\intercal\mathbf{X}(\boldsymbol{\beta}^* - \widehat{\boldsymbol{\beta}}))
\end{aligned}
$$

Similarly, it also holds that

$$
\begin{aligned}
&(\theta^* - \widehat{\theta})(\mathbf{X}\widehat{\boldsymbol{\gamma}} - \mathbf{X}\boldsymbol{\gamma}^*)^\intercal\mathbf{X}\boldsymbol{\gamma}^*(\theta^* - \widehat{\theta}) \\
&= o_p(\|\mathbf{X}\boldsymbol{\gamma}^*(\theta^* - \widehat{\theta})\|_2^2)
\end{aligned}
$$

and

$$
\begin{aligned}
&\|(\mathbf{X}\widehat{\boldsymbol{\gamma}} - \mathbf{X}\boldsymbol{\gamma}^*)(\theta^* - \widehat{\theta})\|_2^2 \\
&= o_p(\|\mathbf{X}\boldsymbol{\gamma}^*(\theta^* - \widehat{\theta})\|_2^2).
\end{aligned}
$$

So we have

$$
\begin{aligned}
&\frac{1}{n}\|\mathbf{X}\widehat{\boldsymbol{\gamma}}(\theta^* - \widehat{\theta}) + \mathbf{X}(\boldsymbol{\beta}^* - \widehat{\boldsymbol{\beta}})\|_2^2 && (13) \\
&= \frac{1}{n}\|\mathbf{X}\boldsymbol{\gamma}^*(\theta^* - \widehat{\theta}) + \mathbf{X}(\boldsymbol{\beta}^* - \widehat{\boldsymbol{\beta}})\|_2^2 + o_p(\frac{1}{n}\|\mathbf{X}\boldsymbol{\gamma}^*(\theta^* - \widehat{\theta}) + \mathbf{X}(\boldsymbol{\beta}^* - \widehat{\boldsymbol{\beta}})\|_2^2). && (14)
\end{aligned}
$$

Recall the restricted eigenvalue condition: there exists a constant $\phi_{0_\beta}^2$ such that

$$
\frac{1}{n\phi_{0_\beta}^2}||(\mathbf{X}\boldsymbol{\gamma}^*, \mathbf{X})\Delta||_2^2 \geq \|\Delta_{S_0'}\|_2^2
$$

for all Δ satisfying $\|\Delta_{S_0'^c}\|_1 \leqslant 3\|\Delta_{S_0'}\|_1$. Plugging in $\Delta = (\theta^* - \widehat{\theta}, \boldsymbol{\beta}^* - \widehat{\boldsymbol{\beta}})$ to the compatibility assumption (the condition $\|\Delta_{S_0'^c}\|_1 \leqslant 3\|\Delta_{S_0'}\|_1$ is satisfied from Lemma 1), together with (14), we have

$$
\frac{1}{n\phi_{0_\beta}^2}\|\mathbf{X}\boldsymbol{\gamma}^*(\theta^* - \widehat{\theta}) + \mathbf{X}(\boldsymbol{\beta}^* - \widehat{\boldsymbol{\beta}})\|_2^2 \geq \|(\theta^* - \widehat{\theta}, \boldsymbol{\beta}^*_{S_0'} - \widehat{\boldsymbol{\beta}}_{S_0'})\|_2^2.
$$

Thus, we have proved $\|(\theta^*-\widehat{\theta},\boldsymbol{\beta}^*_{S_0'}-\widehat{\boldsymbol{\beta}}_{S_0'})\|_1^2\leq(s_{0_\beta}+1)\|(\theta^*-\widehat{\theta},\boldsymbol{\beta}^*_{S_0'}-\widehat{\boldsymbol{\beta}}_{S_0'})\|_2^2=O(\frac{s_{0_\beta}+1}{n}\|\widehat{\mathbf{m}}(\theta^*-\widehat{\theta})+\mathbf{X}(\boldsymbol{\beta}^*-\widehat{\boldsymbol{\beta}})\|_2^2)$.

Finally, we have

$$
\begin{aligned}
&2\sqrt{\frac{1}{n}\|\widehat{\mathbf{m}}(\theta^*-\widehat{\theta})+\mathbf{X}(\boldsymbol{\beta}^*-\widehat{\boldsymbol{\beta}})\|_2^2\cdot\frac{\lambda_\beta}{2}\|(\theta^*-\widehat{\theta},\boldsymbol{\beta}^*-\widehat{\boldsymbol{\beta}})\|_1}\\
\leq&\frac{1}{n}\|\widehat{\mathbf{m}}(\theta^*-\widehat{\theta})+\mathbf{X}(\boldsymbol{\beta}^*-\widehat{\boldsymbol{\beta}})\|_2^2+\frac{\lambda_\beta}{2}\|(\theta^*-\widehat{\theta},\boldsymbol{\beta}^*-\widehat{\boldsymbol{\beta}})\|_1\\
=&\frac{1}{n}\|\widehat{\mathbf{m}}(\theta^*-\widehat{\theta})+\mathbf{X}(\boldsymbol{\beta}^*-\widehat{\boldsymbol{\beta}})\|_2^2+\frac{\lambda_\beta}{2}\|\widehat{\boldsymbol{\beta}}_{S_0'^c}\|_1+\frac{\lambda_\beta}{2}(|\theta^*-\widehat{\theta}|+\|\boldsymbol{\beta}^*_{S_0'}-\widehat{\boldsymbol{\beta}}_{S_0'}\|_1)\\
\leq&2\lambda_\beta(|\theta^*-\widehat{\theta}|+\|\boldsymbol{\beta}^*_{S_0'}-\widehat{\boldsymbol{\beta}}_{S_0'}\|_1)\\
=&O_p(\lambda_\beta\sqrt{(s_{0_\beta}+1)/n}\|\widehat{\mathbf{m}}(\theta^*-\widehat{\theta})+\mathbf{X}(\boldsymbol{\beta}^*-\widehat{\boldsymbol{\beta}})\|_2),
\end{aligned}
$$

where the second inequality holds due to Lemma 1.

Therefore, on set S, we obtain $\|(\theta^*-\widehat{\theta},\boldsymbol{\beta}^*-\widehat{\boldsymbol{\beta}})\|_1=O_p(\lambda_\beta(s_{0_\beta}+1))$ for $\lambda_\beta=O(\lambda_\gamma s_{0_\gamma}^2\vee\sqrt{\log p/n})$.

3.1.2 Proof of the variable selection consistency

Let $\min_{j\in S_0'}|\beta_j^*|=C_1'$, and we define the thresholding estimator $\widehat{\beta}_{\text{thres},j}=\widehat{\beta}_j\mathbb{1}(|\widehat{\beta}_j|\geq C_2')$. Then in terms of variable selection consistency, it means for any j, the sign $(\widehat{\beta}_{\text{thres},j})=\text{sign}(\beta_j^*)$ has probability converges to 1, where sign$(\cdot)$ maps positive entry to 1, negative entry to -1 and zero to zero. Now we are considering two cases. The first case is that if the true signal is 0, then the threshold C_2' has to be big enough so the estimated signal would shrink to 0 as well. Hence for any j such that $\beta_j^*=0$,

$$|\widehat{\beta}_{\text{thres},j}-\beta_j^*|\leq\|\widehat{\boldsymbol{\beta}}-\boldsymbol{\beta}^*\|_1\leq 4\lambda_\beta s_{0_\beta}/\phi_{0_\beta}^2. \tag{15}$$

Hence we require $C_2'\geq 4\lambda_\beta s_{0_\beta}/\phi_{0_\beta}^2$, then $\widehat{\beta}_{\text{thres},j}$ would be 0 as well. On the other hand, for any j such that $\beta_j^*\neq 0$, we want the threshold to be small so $\widehat{\beta}_{\text{thres},j}$ won't be compressed to 0. WLOG, here, we consider that $\beta_j^*>0$ and require that

$$|\widehat{\beta}_{\text{thres},j}-\beta_j^*|\leq\|\widehat{\boldsymbol{\beta}}-\boldsymbol{\beta}^*\|_1\leq 4\lambda_\beta s_{0_\beta}/\phi_{0_\beta}^2. \tag{16}$$

Then, $\widehat{\beta}_{\text{thres},j}\geq\beta_j-4\lambda_\beta s_{0_\beta}/\phi_{0_\beta}^2$, and we do not want $\widehat{\beta}_{\text{thres},j}$ to be 0. So $\widehat{\beta}_{\text{thres},j}\geq\beta_j-4\lambda_\beta s_{0_\beta}/\phi_{0_\beta}^2>C_2'$ for any j, therefore $C_1'>8\lambda_\beta s_{0_\beta}/\phi_{0_\beta}^2$. Finally, we require that $C_2'\geq 4\lambda_\beta s_{0_\beta}/\phi_{0_\beta}^2$ and $C_1'\geq 8\lambda_\beta s_{0_\beta}/\phi_{0_\beta}^2$ to have a variable selection consistency of $\boldsymbol{\beta}^*$. A similar proof can be made for $\beta_j^*<0$. □

3.2 Auxiliary results

Lemma 1. *If the event S holds and $\lambda\geq 2\lambda_{01}$, then* $\frac{2}{n}\|\widehat{\mathbf{m}}(\theta^*-\widehat{\theta})+\mathbf{X}(\boldsymbol{\beta}^*-\widehat{\boldsymbol{\beta}})\|_2^2+\lambda_\beta\|\widehat{\boldsymbol{\beta}}_{S_0'^c}\|_1\leq 3\lambda(|\theta^*-\widehat{\theta}|+\|\boldsymbol{\beta}^*_{S_0'}-\widehat{\boldsymbol{\beta}}_{S_0'}\|_1)$.

Proof. Recall the inequality (10)

$$\begin{aligned}
&\frac{1}{n}\|\widehat{\mathbf{m}}(\theta^* - \widehat{\theta}) + \mathbf{X}(\boldsymbol{\beta}^* - \widehat{\boldsymbol{\beta}})\|_2^2 \\
&\leqslant \frac{2}{n}(\widehat{\mathbf{m}}(\theta^* - \widehat{\theta}) + \mathbf{X}(\boldsymbol{\beta}^* - \widehat{\boldsymbol{\beta}}))^\intercal((\mathbf{X}\boldsymbol{\gamma}^* - \widehat{\mathbf{m}})\theta^* + \boldsymbol{\delta}\theta^* + \boldsymbol{\varepsilon}) + \lambda_\beta\|\boldsymbol{\beta}^*\|_1 - \lambda_\beta\|\widehat{\boldsymbol{\beta}}\|_1.
\end{aligned}$$

Since $\frac{2}{n}(\widehat{\mathbf{m}}(\theta^* - \widehat{\theta}) + \mathbf{X}(\boldsymbol{\beta}^* - \widehat{\boldsymbol{\beta}}))^\intercal((\mathbf{X}\boldsymbol{\gamma}^* - \widehat{\mathbf{m}})\theta^* + \boldsymbol{\delta}\theta^* + \boldsymbol{\varepsilon}) = O_p(\lambda|\theta^* - \widehat{\theta}| + \lambda\|\boldsymbol{\beta}^* - \widehat{\boldsymbol{\beta}}\|_1)$, we have

$$\frac{2}{n}\|\widehat{\mathbf{m}}(\theta^* - \widehat{\theta}) + \mathbf{X}(\boldsymbol{\beta}^* - \widehat{\boldsymbol{\beta}})\|_2^2 \leq c_0\lambda|\theta^* - \widehat{\theta}| + c_0\lambda\|\boldsymbol{\beta}^* - \widehat{\boldsymbol{\beta}}\|_1 + 2\lambda\|\boldsymbol{\beta}^*\|_1 - 2\lambda\|\widehat{\boldsymbol{\beta}}\|_1$$

for some positive constant c. Without loss of generality, we can assume $c_0 = 1$, since we can take $c_0\lambda$ as the new λ if $c_0 \neq 1$. Reorganizing the above inequality, we have

$$\begin{aligned}
&\frac{2}{n}\|\widehat{\mathbf{m}}(\theta^* - \widehat{\theta}) + \mathbf{X}(\boldsymbol{\beta}^* - \widehat{\boldsymbol{\beta}})\|_2^2 + \lambda\|\widehat{\boldsymbol{\beta}}_{S_0'^c}\|_1 \\
&\leq \lambda|\theta^* - \widehat{\theta}| + \lambda\|\boldsymbol{\beta}^* - \widehat{\boldsymbol{\beta}}\|_1 + 2\lambda\|\boldsymbol{\beta}^*\|_1 - 2\lambda\|\widehat{\boldsymbol{\beta}}\|_1 + \lambda\|\widehat{\boldsymbol{\beta}}_{S_0'^c}\|_1 \\
&= \lambda|\theta^* - \widehat{\theta}| + \lambda\|\boldsymbol{\beta}^*_{S_0'} - \widehat{\boldsymbol{\beta}}_{S_0'}\|_1 + \lambda\|\widehat{\boldsymbol{\beta}}_{S_0'^c}\|_1 + 2\lambda\|\boldsymbol{\beta}^*\|_1 - 2\lambda\|\widehat{\boldsymbol{\beta}}_{S_0'}\|_1 - \lambda\|\widehat{\boldsymbol{\beta}}_{S_0'^c}\|_1 \\
&\leq \lambda|\theta^* - \widehat{\theta}| + 3\lambda\|\boldsymbol{\beta}^*_{S_0'} - \widehat{\boldsymbol{\beta}}_{S_0'}\|_1 \\
&=3\lambda(|\theta^* - \widehat{\theta}| + \|\boldsymbol{\beta}^*_{S_0'} - \widehat{\boldsymbol{\beta}}_{S_0'}\|_1).
\end{aligned}$$

□

Proposition 3.2. *The thresholded Lasso estimators* $\widehat{\gamma}_{\text{thres},j} = \widehat{\gamma}_j\mathbb{1}(|\widehat{\gamma}_j| \geq C_2)$ *with* $C_2 \geq 4\lambda_\gamma s_{0_\gamma}/\phi^2_{0_\gamma}$ *satisfy* $P(\text{sign}(\widehat{\boldsymbol{\gamma}}_{\text{thres}}) = \text{sign}(\boldsymbol{\gamma}^*)) \to 1$ *as* $N \to \infty$, *where* sign $(\cdot)$ *is the sign function that maps a positive entry to 1, a negative entry to -1, and zero to zero.*

With a similar argument, we can also argue that the variable selection consistency by thresholded lasso holds for $\boldsymbol{\gamma}^*$.

Let $\min_{j\in S_0}|\gamma_j^*| = C_1$, and we define the thresholding estimator $\widehat{\gamma}_{\text{thres},j} = \widehat{\gamma}_j\mathbb{1}(|\widehat{\gamma}_j| \geq C_2)$. Then in terms of variable selection consistency, it means for any j, the sign $(\widehat{\gamma}_j)$=sign(γ_j^*) has probability converges to 1, where sign$(\cdot)$ maps positive entry to 1, negative entry to -1 and zero to zero. Now we are considering two cases. The first case is that if the true signal is 0, then the threshold C_2 has to be big enough so the estimated signal would shrink to 0 as well. Hence for any j such that $\gamma_j^* = 0$,

$$|\widehat{\gamma}_{\text{thres},j} - \gamma_j^*| \leq \|\widehat{\boldsymbol{\gamma}} - \boldsymbol{\gamma}^*\|_1 \leq 4\lambda_\gamma s_{0_\gamma}/\phi^2_{0_\gamma}. \tag{17}$$

Hence we require $C_2 \geq 4\lambda_\gamma s_{0_\gamma}/\phi^2_{0_\gamma}$, then $\widehat{\gamma}_{\text{thres},j}$ would be 0 as well. On the other hand, for any j such that $\gamma_j^* \neq 0$, we want the threshold to be small so $\widehat{\gamma}_{\text{thres},j}$ won't be compressed to 0. WLOG, here, we consider that $\gamma_j^* > 0$ and require that

$$|\widehat{\gamma}_{\text{thres},j} - \gamma_j^*| \leq \|\widehat{\boldsymbol{\gamma}} - \boldsymbol{\gamma}^*\|_1 \leq 4\lambda_\gamma s_{0_\gamma}/\phi^2_{0_\gamma}. \tag{18}$$

Then, $\widehat{\gamma}_{\text{thres},j} \geq \gamma_j - 4\lambda_\gamma s_{0_\gamma}/\phi^2_{0_\gamma}$, and we do not want $\widehat{\gamma}_{\text{thres},j}$ to be 0. So $\widehat{\gamma}_{\text{thres},j} \geq \gamma_j - 4\lambda_\gamma s_{0_\gamma}/\phi^2_{0_\gamma} > C_2$ for any j, therefore $C_1 > 8\lambda_\gamma s_{0_\gamma}/\phi^2_{0_\gamma}$. Finally, we require that $C_2 \geq 4\lambda_\gamma s_{0_\gamma}/\phi^2_{0_\gamma}$ and $C_1 \geq 8\lambda_\gamma s_{0_\gamma}/\phi^2_{0_\gamma}$ to have a variable selection consistency of $\boldsymbol{\gamma}^*$. A similar proof can be made for $\gamma^*_j < 0$.

References

Bickel, P. J., Ritov, Y., and Tsybakov, A. B. (2009). Simultaneous analysis of lasso and dantzig selector. *Annals of Statistics*, 37(4):1705–1732.

Bühlmann, P. and Van De Geer, S. (2011). *Statistics for high-dimensional data: methods, theory and applications*. Springer Science & Business Media.

Guo, Z., Kang, H., Tony Cai, T., and Small, D. S. (2018). Confidence intervals for causal effects with invalid instruments by using two-stage hard thresholding with voting. *Journal of the Royal Statistical Society Series B: Statistical Methodology*, 80(4):793–815.

Zhang, C.-H. and Huang, J. (2008). The sparsity and bias of the lasso selection in high-dimensional linear regression. *Annals of Statistics*, 36(4):1567–1594.

Zhang, C.-H. and Zhang, S. S. (2014). Confidence intervals for low dimensional parameters in high dimensional linear models. *Journal of the Royal Statistical Society: Series B: Statistical Methodology*, pages 217–242.
